# "A renewed sense of purpose": Mothers' and fathers' experience of having a child following a recent stillbirth

**DOI:** 10.1186/s12884-014-0423-x

**Published:** 2014-12-19

**Authors:** Louise Campbell-Jackson, Jessica Bezance, Antje Horsch

**Affiliations:** Oxford Institute of Clinical Psychology Training, Isis Education Centre, Warneford Hospital, Oxford, OX3 7JX UK; Department of Child and Adolescent Psychiatry, Research Unit, Rue du Bugnon 25 A, University Hospital Lausanne, CH-1011 Lausanne, Switzerland; Department of Neonatology, University Hospital Lausanne, Avenue Pierre-Decker 2, CH-1011 Lausanne, Switzerland; Department of Obstetrics and Gynecology, University Hospital Lausanne, Avenue Pierre-Decker 2, CH-1011 Lausanne, Switzerland

**Keywords:** Stillbirth, Mothers, Fathers, Perinatal loss, Grief, Qualitative research, IPA

## Abstract

**Background:**

Most research has focused on mothers’ experiences of perinatal loss itself or on the subsequent pregnancy, whereas little attention has been paid to both parents’ experiences of having a child following late perinatal loss and the experience of parenting this child. The current study therefore explored mothers’ and fathers' experiences of becoming a parent to a child born after a recent stillbirth, covering the period of the second pregnancy and up to two years after the birth of the next baby.

**Method:**

In depth interviews were conducted with 7 couples (14 participants). Couples were eligible if they previously had a stillbirth (after 24 weeks of gestation) and subsequently had another child (their first live baby) who was now under the age of 2 years. Couples who had more than one child after experiencing a stillbirth and those who were not fluent in English were excluded. Qualitative analysis of the interview data was conducted using Interpretive Phenomenological Analysis.

**Results:**

Five superordinate themes emerged from the data: Living with uncertainty; Coping with uncertainty; Relationship with the next child; The continuing grief process; Identity as a parent. Overall, fathers' experiences were similar to those of mothers', including high levels of anxiety and guilt during the subsequent pregnancy and after the child was born. Coping strategies to address these were identified. Differences between mothers and fathers regarding the grief process during the subsequent pregnancy and after their second child was born were identified. Despite difficulties with bonding during pregnancy and at the time when the baby was born, parents' perceptions of their relationship with their subsequent child were positive.

**Conclusions:**

Findings highlight the importance of tailoring support systems not only according to mothers' but also to fathers' needs. Parents’, and particularly fathers', reported lack of opportunities for grieving as well as the high level of anxiety of both parents about their baby's wellbeing during pregnancy and after birth implies a need for structured support. Difficulties experienced in bonding with the subsequent child during pregnancy and once the child is born need to be normalised.

## Background

Stillbirth is a unique bereavement, which continues to remain a disenfranchised loss [1]. The experience of stillbirth is complex and comprises many losses [2], including loss of future plans, identity as a parent and loss of control [3,4]. Psychological difficulties experienced by mothers include anxiety, depression, negative well-being and grief, which can last up to three years [5,6].

Changes in the categorisation of causes of stillbirth have reduced the number of unexplained stillbirths from 50% to 23% [7]. However, the unexplained category continues to remain the largest. A high number of stillbirths occur in pregnancies that have not had complications [8] and in most cases the loss is very sudden [9]. With no clear answers mothers are left with feelings of shame, guilt and self-blame for the death of their child [10]. Mothers may feel excluded from social groups that they were part of before their loss adding to a sense of isolation and shame [11]. In comparison to other types of child loss, stillbirth is often treated differently in our society and seen as less significant [12], which again may result in profound feelings of shame and guilt [10]. Recent studies have explored the relationship between self-blame, shame and guilt and parental mental health after perinatal loss. Cacciatore et al. [10] conducted an Internet questionnaire-based study with 2, 232 women who had experienced a stillbirth up to three years previously. They identified that 24.6% of women reported blaming themselves and found significant correlations between self-blame and depressive and anxious symptoms. Guilt and shame have been identified as predictors of maternal and paternal grief [13,14]. Barr [14] researched couples after perinatal loss and reported that fathers were more prone to survivor guilt, which in turn impacted on women’s grief.

When compared to mothers, fathers report fewer grief and anxiety symptoms [15]. However, rather than being less intense, fathers' expression of their grief may be qualitatively different from that of the mother [16]. For example, their emotional reaction to the loss may be delayed because of practical demands, such as making funeral arrangements, providing the household income, and a need to remain strong, so as to not burden their partner with their own distress [17]. Despite different grieving styles of parents potentially triggering conflict and affecting the couple relationship [15], a recent study found that most parents thought the stillbirth had strengthened their relationship [16].

Fifty percent of women conceive again less than a year after their loss [18]. This experience is often one of worry, fear of a future loss and a sense of failure [19], in stark contrast to the expected experience of joy and hopeful anticipation. In their subsequent pregnancy, women can experience high levels of anxiety, depression and posttraumatic stress when compared to controls, which in turn may impact negatively on pregnancy outcome, foetal development, and child behaviour [20-23].

The role of the father during the subsequent pregnancy has been highlighted as the provision of support to the mother [24], and fathers have expressed feelings of frustration and helplessness because of their inability to protect their partner from emotional pain [25]. However, fathers have stressed that their feelings of anguish and grief are often not recognised in society and that they often felt ignored and invalidated by their support network and care providers [24,26,27]. Fathers have also described their ambiguity of fatherhood after having lost their baby as another source of distress, in addition to trying to assume a fathering role while grieving [28].

How a previous stillbirth may impact on the maternal-foetal attachment relationship is not yet well understood and conflicting results have emerged. One study reported higher levels of anxiety and decreased prenatal attachment compared to women without a history of loss, whereas another study reported no differences in prenatal attachment between those two groups [29,30].

Infants born after a previous stillbirth are more likely to have disorganised attachments with the main mediating factor being maternal unresolved grief [31]. Reasons for such difficulties have been attributed to 'replacement child syndrome' and 'vulnerable child syndrome' [2,32,33]. It has been suggested that the next born child might reawaken feelings about the previous child, which affects maternal current behaviour. There is some empirical support for the difficulties described in the 'replacement child' and 'vulnerable child' syndromes, but qualitative research into parent’s perceptions of their next born child is needed [34].

Most research has focused on mothers’ experiences of perinatal loss itself or on the subsequent pregnancy, whereas little attention has been paid to both parents’ experiences of having a child following late perinatal loss and the experience of parenting this child [35]. The current study therefore explored mothers’ and fathers' experiences of becoming a parent to a child born after a recent stillbirth, covering the period of the second pregnancy and up to two years after the birth of the next baby.

## Method

### Theoretical basis

Interpretative Phenomenological Analysis (IPA) was chosen because its aim is to understand the specific experiences of individuals in certain circumstances [36,37]. IPA allows the researcher to describe and understand the processes by which an experience is understood [38]. It is concerned with an individual’s subjective report, rather than formulating an objective account and is therefore considered phenomenological [39]. Furthermore, it is recognised as a dynamic process, within which the researcher plays an active role by taking an ‘insider’s perspective’ to explore the essence of the participant’s experience and to explore new areas of knowledge [40]. IPA has been used frequently in psychological research because of its potential to capture concepts that may not have been previously identified by the researcher [41].

### Study design and participants

Twenty-one mothers who had taken part in a previous study, investigating mental health problems in mothers following a stillbirth, and who had provided consent to being contacted about future research, and their partners, were approached via an invitation letter including an information sheet and a consent form. Couples were eligible if they previously had a stillbirth (after 24 weeks of gestation) and subsequently had another child (their first live baby) who was now under the age of 2 years. Couples who had more than one child after experiencing a stillbirth and those who were not fluent in English were excluded. Of the 10 couples who responded with an interest in participating, seven couples (14 participants) met the eligibility criteria and were included in the study.

All interviews were conducted by the first author and took place in participants’ homes. Parents were offered the opportunity to debrief after the interview [42], and given a resource pack of relevant support networks and resources in case they wished to access help. Ethical approval was granted by the Oxfordshire NHS ethics committee (study number: 11/SC/0283).

All interviews were audio recorded with the participants’ informed written consent and lasted between one to two hours. Each partner was interviewed separately. The interviews were semi-structured and aimed to elicit information about the participants' experience of pregnancy, becoming a parent and their relationship with their child born subsequent to stillbirth and their partner. Feedback on the study design and interview schedule was obtained from relevant organisations representing professionals working with this population. Demographic information was also obtained.

### Analytic procedure

Interviews were transcribed, anonymised, and participant codes added in order to ensure confidentiality and privacy of participants. Mothers' and fathers' transcripts were analysed separately in line with the method set out by Smith and colleagues [36]. After reading and re-reading the transcripts to allow immersion in the data set, a detailed set of notes was compiled for each transcript (using descriptive, linguistic and conceptual comments). Emergent themes were detailed and each participant's emergent themes were loosely grouped together. Finally, emergent themes across the data set were formed and superordinate and subordinate themes identified.

Reflexivity, a process that helps to identify the researcher's preconceptions, is an important part of qualitative research [43]. In the current study, the first author was interviewed prior to beginning the study and after analysis to elicit their preconceptions about the research, and subsequently to identify what had been learnt. To enhance transparency, a reflective diary was used throughout the research process to record observations, thoughts and reflections and to help reduce the likelihood of biasing the results [44,45].

To ensure methodological rigour and credibility, procedures recommended for the publication of qualitative research in psychology were followed [46]. Peer validation was used rather than participant validation due to the interpretative nature of IPA, which may make participant validation counter-productive [47,48]. Credibility checks were achieved through triangulation with members of the research team (AH, JB). All stages of analysis were discussed within regular peer support meetings.

## Results

### Participants

All parents were married. Five mothers and six fathers were British, one mother and one father were Polish and one mother Brazilian. The mean age of parents was 31.9 years (SD = 3.5). All participants were employed at the time of interview. The mean gestational age at the time of stillbirth was 36.1 weeks (SD = 4.6) and the mean age of the child born subsequently was 16.6 months (SD = 7.4). Three couples had received bereavement counselling and two mothers had medication for postnatal depression following their stillbirth.

### Superordinate and subordinate themes

Five superordinate and fourteen subordinate themes emerged from the data, covering the experience of the second pregnancy and becoming a parent to a live baby following a previous stillbirth (Table 1). The first superordinate theme details the parents’ experience of uncertainty throughout their pregnancy with their second child and the second superordinate theme, the strategies they utilised to cope during this time. The third superordinate theme explores parents' relationship with their second child from pregnancy until present day and the fourth superordinate theme depicts the on-going grief process and its impact on the couple’s relationship. The final superordinate theme explores the experience of becoming a parent, how they understand this role and the impact of others on this experience. Superordinate and subordinate themes will be discussed in turn using direct quotations as illustrations. Additional quotes to the ones presented in the text are presented in Table 1.

**Table 1:** **Superordinate and subordinate themes**

**Superordinate themes**	**Subordinate themes**
**1. Living with uncertainty**	**1.1. Expecting the worst**
	Mother: *I had in the back of my head stillbirth is related to cot death .., so I had in my head that he was going to have, you know, he was going to die in his cot, in his sleep and he got jaundice for a week and I panicked, I thought that’s it, that’s gonna kill him too, and then he got the snuffles and I thought he’s gonna die in his snot (laughing). I was just so paranoid the whole time. You know this is his last day, this is his last week. (M6, 502)*
	Father: *I feel like very anxious now, about something happening to (second child), or becoming ill, or losing him which I guess counselling would help with. It does feel quite, I wouldn’t have had that before. We are very anxious towards his health, probably a bit over the top. (F4, 164)*
	**1.2. Staying strong**
	Mother: *Because I thought the way I felt was so huge, absolutely enormous, I just thought I just didn’t want, until I was separate from (second child), you know I didn’t I just didn’t want to express anything at all, so I just held (laughing) which probably isn’t that healthy. (M6, 123)*
	Father: *It wasn’t my job to worry. It was my job to look after (wife). I sort of tried to put it to the back of mind and think about it a little bit, and I wouldn’t say it would go away but just deal with the problems. (F5, 196)*
	**1.3. A process of acceptance**
	Mother: *Part of me still thinks when I go in a check him in the morning, is he still alive and that’s difficult. I don’t think I’ll ever get over that. Its got easier as he’s got bigger as especially the risk of cot death is much lower now he’s 9 months.” (M5, 577)*
	Father: *So in that respect he’s taught us that he’ll survive, so now that what we’ve learnt from him. Day by day just, he’ll be alright and we should stop worrying. (F7, 363)*
**2. Coping with uncertainty**	**2.1. Cognitive strategies**
	Mother: *It was just like we’d have a scan and we’d build up, you’d build up for the scan and you know I’ll be crying the night, a few nights before and hysterical or whatever … and they’re gonna say he’s dead or this has happened or something’s not right … and then we’d go and everything would be fine and you just had this big sense of relief … then you’d come home and I’d probably get maybe three days of relative calm and then it would start again.* (M4, 508)
	Father: *I remember just thinking we’ve got to get through this, we’ve got to get through this. (F3, 241)*
	**2.2. Emotional strategies**
	Mother: *You kind of try and fight, you can’t, with a baby growing inside of you, you can’t direct any of that to it and you wouldn’t want to and so you just kind of feel like you’re pushing like any anxiety to anything else anything that you definitely can. (M3, 100)*
	Father: *I sort of stopped myself thinking that it was happening in a way with (second child). Because it was sort of like don’t get too involved this time, in case it goes wrong again. (F1, 53)*
	**2.3. Practical strategies**
	Mother: *I’ve got a mirror in the car where I can see, in my rear view mirror I can see (second child). Because he’s rear facing. So to start off with when I was driving around I couldn’t see him and he’d be asleep and I hated it. So I got one of these mirrors that move so you can actually see him. (M, 558)*
	Father: *I’ve got a new job and that’s a good thing it makes me happy and keeps my mind busy and occupied. It’s a form or escapism. (F7, 87)*
**3. Relationship with the next child**	**3.1. Bonding with the next child**
	Mother: *You need to think of a name and all of that, and you do that, but at the back of your mind, I was thinking right OK, I need to have an outfit for him to go home in, but it also needs to be an outfit that he could use, if he needs to be buried in it. (M5, 137)*
	Father: *After (second child) was born, within the first few weeks and months, I had a hard time connecting with her. I knew that I loved her and I was chuffed but I didn’t feel like, it was just ok, and when they handed her to me when she was born, I had this feeling that I would have this sense of elation and I would be so relieved to finally have a baby, but I didn’t get that, I feel bad saying it, but it was like there was a kind of numb. (F1,140)*
	**3.2. Changed priorities**
	Mother: *Everyone else was stressing about sleepless nights and there’s me and you know we’ve spent night, we’ve spent nights and nights being awake crying you know and being upset and can’t sleep because we’ve lost our baby like, sleepless nights will be fine. (M4, 359)*
	Father: *We would rather our lives revolve around him as opposed to he sort of fits into our lives. Which is good and bad I guess, we can’t help it. He’s like everything to us*. (F7 75)
**4. The continuing grief process**	**4.1. Challenges in attending to grief**
	Mother: *You’re so wrapped up in the new arrival, and it’s all new to you and … I suppose in a way, I was very much like any new mum, with a baby. I wrapped myself up so much with (second child) and what was going on, maybe I did that on purpose in a way. Because I wanted to make sure everything was absolutely with (second child) and that we were doing the best we could for (second child). I didn’t let too many emotions go towards (first child). (M1, 333)*
	Father: *I had to spend a lot of time reassuring and trying to be stronger and I wouldn’t say it was to the exclusion of being able to grieve… buts it’s a heavy burden I suppose to carry, carry two lots because there is fear as well. (F6, 24)*
	**4.2. Joy and grief in parenting**
	Mother: *It just, it reminds you of all those times, and thinking like the first bath, you do with (second child), you’re reminded of the fact that you’re really sad that you didn’t do the first bath with (first child)… and it makes you feel bad, because you’re like, but you’re here, and I do love you, but I would have liked to have had this time with your brother.* (M3, 700)
	Father: *He did look a lot like his sister and that was both beautiful and hard in the same way. Because you just think wouldn’t it be perfect if she was here now as well.* (F6, 288)
	Mother: *People assumed that it would make it all better because you we’re pregnant again. Oh, we can forget about (first child) now, you know. That’s in the past, you’re having another baby, isn’t that nice, let’s focus on that. And I felt that was really difficult. Because it was almost like (first child) was forgotten and everyone’s focus was on this new baby. (M1, 466)*
	Father: *It would be an injustice to (first child) to just move on and forget about it. So you kind of want to hold on to a bit of the grief I suppose. (F1 697)*
	**4.3. Remembering the lost child**
	**4.4. Impact of grief on relationships**
	Mother: *I don’t think we would have resolved it between ourselves. I think a third party. They just forced him into talking, he didn’t want to, but she really, she was such a good counsellor and she really you know, helped us understand each other and where we were both coming from. (M4, 770)*
	Father: *That’s maybe the female approach as opposed to the male approach. Always asking why and what (regarding the stillbirth). If I can’t answer the question why are we talking about it?* (F7,454)
**5. Identity as a parent**	**5.1. Impact of self-blame on parenting**
	Mother: *A lot of the time I was thinking, maybe (first child) died for a reason; maybe it was because I was going to be a bad mother.* (M5, 321)
	Father: *Obviously she’s a little girl it felt like I should be protecting her even though there was nothing you could do. If felt like she’s your child, your little girl, you should have been able to do something or should have known it was going to happen, it felt quite, not like you let them down, but yeah, this it was something you should have done as a father.* (F4, 174)
	**5.2. Fulfilling the role of a parent**
	Mother: *Mother’s day was in the April before I had (second child) and I you know (sighing), we discussed it before hand like Father’s day and we didn’t mark it, because we didn’t feel like that we were actually parents (M5, 590)*
	Father: *We knew we were going to be parents properly this time. (F3, 606)*
	**5.3. Feeling different to other parents**
	Mother: *I felt I could never complain about it ever, because to my family never, because they’d be like, oh you know, oh well you shouldn’t moan you know, 7 months, about a year ago you’d have taken this …I just felt like if I ever moaned about it, people were thinking God what you moaning about, you’re lucky. (M4, 324)*
	Father: *You just become separate somehow and then that makes you become a bit introspective and a bit oh maybe we’re different. (F1, 572)*

#### Superordinate theme 1: Living with uncertainty

All parents described the experience of living with uncertainty about their subsequent child’s physical wellbeing and survival, which was present during pregnancy and continued once the child was born. As their child got older, they gradually came to terms with uncertainty and the worries lessened.

*Subordinate theme 1: Expecting the worst.* All mothers described high levels of anxiety, fear and worry during their subsequent pregnancy. For some mothers, the anxiety was “*debilitating*” and one mother described her subsequent pregnancy as

*…kind of like the worst nine months of my life, other than my experience with (first child) because the worry just drove me to madness. (M3, 33)*

Although all of the fathers described worry and fear throughout pregnancy, some thought that their experience during the time of pregnancy was less intense than their wives’.

*I could ignore it quite well compared to her because I think it’s not happening to me. (F4, 16)*

Most parents reported expecting the worst outcome, which continued after the birth of the live baby. A sense of the fragility of life and uncertainty about their subsequent child’s future emerged, which at times made it difficult to separate from their child. Parents also felt a lack of control during this time. For some, these worries were present daily.

*Subordinate theme 2: Staying strong.* Mothers and fathers reported a need to “*stay strong*” whilst living with uncertainty. Parents took on different roles: for mothers, their focus was on protecting their child and for fathers, their motivation was to protect their partner in addition to their (unborn) child. Fathers described managing their own anxieties whilst providing support for their spouse.

*I had to spend a lot of time reassuring and trying to be stronger and I wouldn’t say it was to the exclusion of being able to grieve… buts it’s a heavy burden I suppose to carry, carry two lots because there is fear as well. (F6, 24)*

*Subordinate theme 3: A process of acceptance.* Most mothers and fathers experienced a process of “*gradually”* coming to terms with uncertainty and the worries lessened as their child got older. Some parents made a conscious decision to overcome their fears and this was spurred on by wanting to be psychologically available for their subsequent child.

#### Superordinate theme 2. Coping with uncertainty

All parents discussed strategies they employed to manage the feelings of uncertainty. These strategies were broadly categorised into three groups: cognitive, practical and emotional strategies.

*Subordinate theme 1: Cognitive strategies.* All parents used cognitive strategies, such as distraction, mainly during pregnancy. For fathers, work was the most reported source of distraction. Some fathers also reported using positive self-talk as a way of reducing their anxiety.

*I was trying to convince myself that everything would be alright. I was very often reassuring myself thinking that is was bad luck we had the first time and that we’ll be alright. (F2, 91)*

*Subordinate theme 2: Emotional strategies.* Mothers and fathers used emotional strategies during pregnancy in order to protect their unborn baby, such as trying to control, ignore or fight their emotions. For some, this continued once their child was born.

*I think just having to keep control of my feelings and my fears and worries because I think I just don’t want to be a person that puts that on to him. (M6, 694)*

*Subordinate theme 3: Practical strategies*. Most parents reported using practical strategies to cope with uncertainty. During pregnancy the main practical strategy employed by mothers was monitoring foetal movements.

*I was eating ice poles sat on the sofa at 3am trying to make him move. Have I felt him move? Then I suddenly went into panic, which I didn’t in my first pregnancy. (M5, 548)*

Reassurance was also sought, predominantly from hospitals. Most parents reported that they found this helpful but for some, reassurance gave only short-term relief.

For most parents, monitoring their child to ensure she/he was still alive continued after she/he was born. Mothers and fathers expressed difficulties separating from their children, which appeared motivated by a desire “*to protect*” them and ensure that no harm would come to them.

#### Superordinate theme 3. Relationship with the next child

All parents described their relationship with their subsequent child from the bonding process in pregnancy until the present day. This also includes the importance of the child in their lives and their changing priorities.

*Subordinate theme 1: Bonding with next child.* Most mothers and fathers described a sense of going against their “*natural instincts”* to attach to their child during pregnancy by delaying or not preparing for their next child.

*I didn’t really want a name or anything until he was here. Because we did that so much with (first child) we knew the name, we did the bedroom, we did all those things and with (second child) I really didn’t want to go there at all. (F4, 304)*

Both mothers and fathers experienced an underlying sense of guilt about the challenge of bonding during pregnancy. If parents reported engaging in bonding behaviours such as playing music to their baby and finding out the sex of their child, this was in preparation for loss rather than life.

When describing their experience of meeting their child for the first time after birth, most parents spoke of an overwhelming sense of “*relief*” that their child had been delivered safely. Some experienced emotional numbness, which overshadowed other expected emotions such as joy. Some mothers described needing time to adjust to the birth of a live baby that they had not been expecting and had not been preparing for.

*I asked the midwife for him not to be put on to me. Because I know it’s important for babies to have skin to skin contact. But I just couldn’t imagine, because I had spent all this time imagining that he doesn’t exist. So he was like a stranger really. I mean a very well loved stranger. (M6, 379)*

For some mothers, initial bonding didn’t feel entirely “*natural*” and they needed to be “*methodical*” at first, in order to feel closer to their child. The process of bonding developed as the child became more communicative/ interactive and all parents stated they now had a good relationship with their subsequent child.

*Subordinate theme 2: Changed priorities.* All parents described a change in their perspectives since the birth of their next-born child. They reported valuing and prioritising time with their child. For example, some fathers adapted their working hours in order to spend more time with their child. They also described having a different perspective on some of the challenges of parenthood, positively viewing situations that might cause most parents to feel stressed.

*We’re so grateful to have her here with us. There’s no point, we’ll, you know, we’ll get our sleep at some point and we’ll overcome whatever little problem there is.* (M1, 468)

For most parents, prioritising time with their child allowed them to “*make up for lost time*” with their previous child. The previous loss also meant that some parents valued time with their child due to continual feelings of uncertainty about their child’s survival.

#### Superordinate theme 4. The continuing grief process

All parents described a continuing experience of grief during the subsequent pregnancy and after their second baby had been born. This process happened in parallel to the parenting process and included the impact of grief on others and the challenges some faced in attending to their grief.

*Subordinate theme 1: Challenges in attending to grief.* Most mothers described challenges in attending to their grief, as they wanted to protect their subsequent child from their feelings of grief during pregnancy and parenting. Once their child was born, they found that having a newborn did not allow the time or space to grieve. Some fathers found it difficult to find space to grieve as they felt they needed to stay “*strong*” in order to support their spouse.

*It was hard because you had to stay strong all the time for (partner's) sake and therefore a lot of things that I emotionally wanted to release … you don’t really get an outlet.* (F6, 16)

*Subordinate theme 2: Joy and grief in parenting.* Most parents described the joys of becoming a parent. However, for both, this was also a greatly “*confusing*” time, as while it was one of immense happiness, they also experienced grief for the child they had lost. For many, the first experiences with their subsequent child served as reminders of their loss.

Most parents highlighted that their next child was not a “*replacement*” and did not alleviate their grief fully.

*There was never a sense of a replacement with me. It was always a case of they were two very separate children and our family includes (first child). (F1, 291)*

Parents reported going through a process of “*coming to terms*” with the fact that they were having a different child and that this gave them a “*renewed sense of purpose*”.

*Subordinate theme 3: Remembering the lost child.* All parents described wanting to remember their baby that had died. One mother explained that by helping others in telling the story of her first child, she was creating his *“legacy”.* Another father explained that his grief maintained a relationship with his stillborn baby and that it felt like an *“injustice”* if he *“moved on”.* Some parents described maintaining a relationship with their stillborn baby through commemorating anniversaries, imagining how they would look now, and through their own grief. One mother encapsulated the dilemma of how to love both of their children in the following extract:

*I imagined that my heart, you know was like an ocean, just completely full of love… and then when I was expecting with (second child) I really struggled thinking will I love one more and love one less, and will (first child) be jealous. I know it sounds a bit odd, but will she look down from heaven and feel jealous, and will I screw (second child) up because of how I feel about (first child) and he’ll feel like he’s filling a hole … The heart is such an amazing thing because it does, you think it can’t get any bigger and it does get bigger and it gets, the capacity to love does get more. (M6, 634)*

Some parents reported difficulties in sharing memories of their first born with other people who expected them to move on now that they had given birth to a subsequent child.

*Subordinate theme 4: Impact of grief on relationships.* Most parents described feeling challenged by other people’s grieving styles, particularly that of their spouses. Gender differences in grieving styles and being at a different stage of grief from their spouse were highlighted as challenges.

*I can see why some people would divorce over such a thing, if it’s not quite as all consuming for them as it is for you, and you don’t understand their reaction, why it doesn’t take over their whole life. (M3, 736)*

Some couples reported that they learned to understand their partner’s way of coping, which for two couples was facilitated by counseling input. However, all parents reported growth in their relationship with their partner since their experience of loss, including greater emotional closeness, better communication, greater “*mutual understanding*”, and a unifying bond.

#### Superordinate theme 5. Identity as a parent

All participants described their identity as a parent. This theme explored feeling as though they had failed previously as parents and its impact on current parenting. Most participants felt that they were finally fulfilling the role of becoming mothers and fathers but with a continued sense of being different from other parents.

*Subordinate theme 1: Impact of self-blame on parenting.* Most parents described self-blame for having had a stillbirth. Because of this sense of self-blame, most mothers reported placing high expectations on themselves as a parent of their second child, encapsulated by wanting to be “*supermum*”. There was a sense of needing to make up for previously failing as a mother.

*There is that added pressure to be a fantastic mummy …and to give him everything that he could possibly need to prove that I would have been a good mum to (first child) … I wanted him to be advanced because if he was advanced that meant that I was doing the right thing.* (M5, 700)

*Subordinate theme 2: Fulfilling the role of a parent.* Most parents described the normative learning curve involved in becoming a parent and the challenges associated with it. Some described having felt like mother/father *“without a child”.*

*Because (first child) died I was completely blank about parenthood…It sounds scary but I’m a mother but I don’t have a child, so I don’t have experience, so my second child would be my first one to practice on it.*(M2, 348)

After having a healthy baby they were able to fulfil not only the role of parent but also the lost dreams of having a child. This meant they were “*not living in the margins of life*” any longer.

*Subordinate theme 3: Feeling different to other parents.* Most parents talked about comparisons between themselves and other parents, which left them feeling “*different*”. A sense of difference made it difficult to discuss their experience and loss history, particularly with other expectant parents in order to protect others’ feelings.

*…one said, are you worried about bringing the baby home. And I remember just looking at her and thinking no I’m worried about not bringing the baby home.* (M4, 334)

Adding to a sense of difference was feeling misunderstood by friends or professionals who lacked experience or knowledge of stillbirth. Compounding the sense of isolation and adding to the pressures of being a new parent, was a feeling the majority of parents described of not being able to voice their difficulties to others. They expressed concern at appearing “*ungrateful*”, as others expecting them to feel grateful made it difficult to discuss the challenges of parenthood.

## Discussion

This is the first study investigating the lived experiences of mothers and fathers of becoming a parent to a child born after a recent stillbirth, covering the period of the second pregnancy and up to two years after the birth of the next baby. Overall, the fathers' experiences were similar to the experiences of mothers. However, some differences were identified between mothers and fathers with regards to the reasons why they controlled their emotions during the subsequent pregnancy and after their second child was born: mothers solely focused on protecting the (un)born child, whilst fathers in addition also took on the role as the main support of their partner during pregnancy. Fathers reported challenges with finding the space to grieve and it appeared that mothers and fathers also expressed their grief qualitatively differently. As the baby continued to live, parents reported that the sense of uncertainty gradually decreased and it appeared that with time, fathers’ roles as the main support for their partners became less necessary, possibly allowing the opportunity to grieve. Furthermore, despite at times feeling challenged by their partner’s grieving style, mothers and fathers reported greater mutual understanding and mutual support within their relationships.

All parents describing experiencing high levels of anxiety during their subsequent pregnancy and after their second child was born is consistent with previous research [27,35]. However, the cognitive and emotional coping strategies mothers and fathers employed to manage living with uncertainty have not been identified previously. Many of the strategies used in pregnancy appeared useful in the short-term but did not reduce their anxiety in the long-term and were sometimes counter-productive. This is in line with evidence showing that safety behaviours such as reassurance seeking and distraction provide only transient relief and can serve to maintain anxiety [49].

After birth, parents continued to employ strategies to manage the threat that their child could become ill or die at any moment, which sometimes led to difficulties in separating. This finding is consistent with the literature [27,35]. Although some parents began a journey of acceptance resulting in a lessening of worries, this was not the case for all, as some felt the threat of death continued.

In line with Philips and Carr’s [50] suggestion of the challenges women experiencing a high risk pregnancy might face, mothers and fathers in our study described limiting or not bonding at all with their unborn child in order to protect themselves, and others, from the distress of a potential future loss. Parents reported delayed or absent “nesting” behaviour, not imagining a future with their child, or denial of the pregnancy. This is the first time this is described in fathers. Furthermore, the concept of parents engaging in some attachment behaviours in preparation for loss rather than birth has not been identified by previous research.

Parents' perceptions of their attachment with their subsequent child were positive at the time of interview. After initial difficulties in developing the attachment relationship, this became more “*natural*” as time went on and their child became more interactive. Although mothers reported feeling guilt for struggling to emotionally attach during pregnancy or immediately after birth, they did not report consciously limiting emotional attachment in order to protect themselves once the baby was born, which is in contrast to previous research [35]. Our finding that fathers experienced guilt in a similar way adds to the existing knowledge base.

All parents provided positive descriptions of their child and did not compare them negatively with the stillborn infant. Most parents described worrying less about the usual challenges of parenting. This is in contrast to previous findings [51] that mothers with a loss history reported a greater number of difficulties with their child’s behaviour. The current findings also conflict with another study [52] in which mothers perceived their second child as less ideal and reported greater difficulties with their babies' routine than controls. However, this was not previously investigated in fathers.

All parents grieved for their first child during the subsequent pregnancy but described challenges in attending to their grief. Mothers felt they could not grieve in order to protect the unborn child and fathers felt they needed to support their partner. This fits with a previous description of the role of the father during the subsequent pregnancy as the provision of support to the mother [24]. Both parents in our study continued grieving after the second baby was born. Although maternal unresolved grief was highlighted in one study [30], this has so far not been looked at in fathers.

For most participants, becoming a parent to a live baby was a confusing time due to conflicting feelings of the joy of parenting but also grief for their first child. However, mothers' and fathers' narratives did not appear to fit with the concept of ‘replacement child syndrome’ [53]. This is in line with an earlier study of mothers [54], but the inclusion of fathers adds to the knowledge base. 'Replacement child' syndrome has been criticised for being based on a small number of clinical cases and not fully explaining the meaning of the lost child for parents [55].

In line with narrative models of loss, parents described changes in how they made sense of their loss [56]. For example, one mother was unsure at first if she could love both (stillborn and subsequently born) children but later realised she could. The findings also show changes in the intensity of grief during different time periods, such as when their subsequent child met certain milestones.

Parents describing ways in which they maintained a relationship with their lost child fits with O’Leary’s [55] suggestion that bonds are not broken but re-ordered to allow new attachments to the subsequent child. Although their priority was their next-born child, parents described that being able to maintain a relationship and to remember their first child with others was important. This was previously described in fathers but not in mothers [27].

Similarly to other studies [57,58], most mothers and fathers reported challenges associated with their partners' differing grieving style. As was found previously, fathers described having to focus on practical tasks and needing to remain strong, which meant that their expression of grief was qualitatively different from that of their partners [59]. However, in line with recent research [16], all couples additionally reported an experience of growth in their relationship with their partner.

Most parents described thoughts of self-blame and guilt for having had a stillbirth, particularly when no medical explanation was provided, which has previously been reported [54]. However, some mothers placing themselves under great pressure as a parent (wanting to be a *supermum*) as a result of the self-blame is a new finding with important implications. This may lead to high levels of parenting stress, which could impact negatively on the quality of parenting and family relationships.

This study has several limitations. All mothers had previously taken part in quantitative research and may therefore represent a group of parents who are particularly articulate. Furthermore, the current study has a cross-sectional design and relies on retrospective reporting. Strengths of the study are that both mothers and fathers were included and that the interview covered the period of the second pregnancy and the transition into becoming a parent for the second time. All interviews were conducted in participants’ homes, thus facilitating an opportunity to talk freely about a sensitive topic [60]. In addition, all authors were involved in the interpretative process, which enhances the authenticity of the accounts generated.

### Clinical implications

Our findings highlight the importance of tailoring support systems not only according to mothers' but also to fathers' needs. Parents’, and particularly fathers' reported lack of opportunities for grieving, suggests that signposting to additional forms of support where opportunities are given to express and work through this may be helpful e.g. support groups or grief counselling. The high level of anxiety and worries of both parents about their baby's wellbeing during pregnancy as well as after birth implies that support should be offered to normalise and contain these fears. O’leary and Thorwick [27] recommend support groups, preferably held in a mainstream setting, which would allow parents time to process the challenges around attachment and grief and also normalise their experiences.

Results also show that mothers' and fathers' difficulties experienced in bonding with the subsequent child during pregnancy and once the child is born may need to be normalised. Some mothers may also need a period of adjustment immediately following labour and obstetric staff should respond sensitively to these needs.

Some parents may require additional support during subsequent pregnancy and the postpartum period to prevent the development of attachment difficulties (O’leary and Thorwick) [27]. Cote-Arsenault et al. [61] outline a model of nursing care for support for women during the subsequent pregnancy- providing a safe and supportive environment through home visits, teaching anxiety management techniques and facilitating diary writing. Further research into interventions supporting both parents is required. Interventions involving mindfulness practices may provide a model of care which is supportive for clients experiencing a traumatic loss, as well as the healthcare professionals providing the support (Cacciatore & Flint) [62]. However, further research is required to evaluate these interventions with this client group.

Feelings of self-blame and guilt were highlighted throughout the findings for both mothers and fathers, suggesting that these emotions may be a normal response Cacciatore et al. [1]. Healthcare professionals need to be aware of self-blame and guilt and the possible impact on parental mental health Cacciatore et al. [1] and parenting style. Professionals need to also be sensitive with their use of language as to not further increase feelings of self-blame (Cacciatore et al. [63], Cacciatore et al. 2014). Barr [14] recommends that bereavement counselling actively attends to feelings of shame and guilt and due to the role of fathers' survivor guilt on women’s grief, couples counselling may be beneficial.

## Conclusions

Findings highlight the importance of tailoring support systems not only according to mothers' but also to fathers' needs. Parents’, and particularly fathers', reported lack of opportunities for grieving as well as the high level of anxiety of both parents about their baby's wellbeing during pregnancy and after birth implies a need for structured support. Difficulties experienced in bonding with the subsequent child during pregnancy and once the child is born need to be normalised. Some parents may require additional support during subsequent pregnancy and the postpartum period to prevent the development of attachment difficulties. Feelings of self-blame and guilt were highlighted for both mothers and fathers and healthcare professionals need to be aware the possible impact on parental mental health and parenting style.
